# CUL4B regulates thyroid cancer differentiation and treatment sensitivity by ubiquitinating ARID1A

**DOI:** 10.1016/j.tranon.2025.102389

**Published:** 2025-04-11

**Authors:** Haiyan Gu, Bo Han, Jing Hu, Ping Liu, Wenyao Liu, Ying Qu, Lin Zhang, Panpan Li, Gongzheng Wang, Zhiyan Liu, Mei Qi, Feifei Sun

**Affiliations:** a Department of Pathology, Qilu Hospital, Shandong University, Jinan 250012, China; b Department of Pathology, The Affiliated Hospital of Qingdao University, Qingdao, China; c Department of Pathology, Peking University People's Hospital, Beijing, China; d Michigan Center for Translational Pathology, University of Michigan, Ann Arbor, MI, USA; e The Key Laboratory of Experimental Teratology, Ministry of Education and Department of Pathology, School of Basic Medical Sciences, Shandong University, Jinan, Shandong, China; f Department of Pharmacy, The Second Hospital, Cheeloo College of Medicine, Shandong University, Jinan, Shandong, China; g Binzhou Center for Disease Control and Prevention, Binzhou, Shandong, China; h Department of Pathology, Shanghai Sixth People's Hospital Affiliated to Shanghai Jiao Tong University School of Medicine, 600# Yishan Rd, Shanghai, China

**Keywords:** CUL4B, Thyroid cancer, Differentiation, ARID1A, MAPK

## Abstract

**Background:**

Thyroid cancer (TC) is a prevalent endocrine malignancy with a generally favorable prognosis. However, dedifferentiation of TC poses a significant challenge, resulting in poorer patient outcomes and necessitating urgent attention. Cullin 4B (CUL4B), a scaffold protein involved in proteolysis and epigenetic regulation, has been reported to play an oncogenic role in many human malignancies, though its involvement in TC remains unclear.

**Methods:**

The association between CUL4B expression and prognosis in TC patients was assessed using immunohistochemistry. RNA-seq was utilized to investigate the underlying molecular mechanisms, which were further validated through *in vitro* experiments. The target gene of CUL4B was identified, and the complete ubiquitination regulation process was described. The phenomenon of high expression of CUL4B in TC was explained by identifying that CUL4B-mediated regulation of the SWI/SNF complex.

**Results:**

Our findings revealed that CUL4B expression was positively correlated with tumor progression and poor prognosis in TC. Mechanistically, overexpression of CUL4B promoted the progression and dedifferentiation of TC *in vivo* models. Crucially, we discovered that CUL4B drives dedifferentiation by promoting the ubiquitination of ARID1A within SWI/SNF complex, leading to decreased expression of the differentiation marker paired box 8 (PAX8). This loss of PAX8 contributes to the dedifferentiation process, ultimately resulting in the formation of anaplastic thyroid carcinoma (ATC). Moreover, silencing CUL4B increased the sensitivity of TC cells to MAPK inhibitors.

**Conclusion:**

CUL4B was crucial in driving tumor advancement and inhibiting differentiation in TC by facilitating the ubiquitin-mediated degradation of ARID1A, underscoring its potential as a therapeutic target.

## Highlights

• In the current study, for the first time, we dissected the role of CUL4B in TC.
• We characterized that CUL4B ubiquitinates and degrades the SWI/SNF complex while competitively binding with ARID1A in the SWI/SNF complex to PAX8 at the transcription initiation site, ultimately leading to the downregulation of PAX8 expression.
• A combination of CUL4B inhibitor, trametinib and dabrafenib may lead to more effective treatment for TC patients.

## Introduction

While papillary thyroid carcinoma (PTC) and follicular thyroid carcinoma (FTC) generally exhibit favorable prognoses with high cure rates, poorly differentiated thyroid carcinoma (PDTC) and anaplastic thyroid carcinoma (ATC)-characterized by dedifferentiation remains notoriously aggressive, associated with treatment resistance and poor outcomes, representing a significant clinical challenge [1].

The molecular mechanisms driving dedifferentiation and chemoresistance in PDTC and ATC require further investigation to develop more effective therapies. Genetic alterations were known to be primary drivers of tumorigenesis and progression in thyroid carcinoma (TC) [[2], [3], [4]]. Identifying specific mutations with prognostic and therapeutic value was essential for developing personalized therapeutic approaches, allowing tailored treatments for individual patients in cancer medicine. For example, SWI/SNF mutations can inhibit TC differentiation and modulate the sensitivity of TC cells to chemotherapy through the regulation of IKKα signaling [5,6]. Similarly, ARID1A, a post-translational regulator of mitogen-activated protein kinase (MAPK) pathway, influences cellular responsiveness to chemotherapy [7,8]. Dysregulation of the MAPK and phosphoinositide 3-kinase (PI3K) pathways frequently resulted in impaired function of the Na+/*I*− symporter (NIS), which was essential for iodine uptake in thyroid follicular cells [9]. Paired box 8 (PAX8), a developmental transcription factor, was crucial for the establishment of follicular thyroid cells in both mice and humans [10,11]. It was also expressed in the developing kidney, neural tube, and thyroid [[12], [13], [14]]. PAX8 plays a pivotal role in regulating NIS transcription and thereby influences iodine uptake in thyroid follicular cells [15,16]. PAX8 was essential for thyroid gland development and the maintenance of its differentiated state [17]. Targeting tumor mutations with therapeutics may be instrumental in overcoming therapeutic resistance in TC. Therefore, continued research into these molecular mechanisms was crucial for improving treatment strategies [18].

Ubiquitination was recognized as a crucial epigenetic regulatory mechanism in the development and progression of various cancers. Cullin 4B (CUL4B), a member of the cullin family, played a significant role in this process [19,20]. The protein products of this family were essential components of Cullin-Ring E3 ubiquitin ligase complexes (CRLs) that can ubiquitinate a wide array of substrates involved in diverse cellular processes, such as cell cycle progression, gene expression, signal transduction, the DNA damage response, and embryonic development [21,22]. CUL4B acted as a scaffold protein within the Cullin4B-Ring E3 ligase (CRL4B) complex, contributing to proteolysis and epigenetic regulation [23]. Recent studies have highlighted CUL4B's overexpression in several malignancies, including tumors of the colon, stomach, liver, and lung, suggesting its involvement in cancer progression [[24], [25], [26], [27]]. Our previous research has identified an oncogenic role for CUL4B in gastric and prostate cancers [28,29]. However, the role of CUL4B in TC has not been thoroughly investigated. Thus, this study aimed to explore the expression and biological functions of CUL4B in TC, with an emphasis on elucidating the mechanisms by which CUL4B enhances TC cell growth, invasion, and dedifferentiation.

## Materials and methods

### Tissue collection and tissue microarrays

A total of 159 PTC, 19 ATC, and 36 normal thyroid cases were collected from the Qilu Hospital of Shandong University (Jinan, China). Follow-up time starts with the day of the initial surgical treatment and finishes with the date of the first relapse (persistence or recurrence cases) or the last follow-up (non-recurrence or dropped-out cases). Follow-up data were available for 146 patients, ranging from 18 to 232 months (mean, 143 months). Tumor recurrence was defined as an active disease in the residual thyroid, lymph node, or distant metastases occurring after 1-year follow-up. It was assessed by the serum thyroglobulin level, ultrasonography, or imaging scans and confirmed by pathological diagnosis on excision or cytology. A total of 3 tissue microarrays (TMAs) were constructed, and duplicate cores (1.0 mm in diameter) were taken from representative tumor areas as previously described. The approval was obtained from the Shandong University ethics committees. Informed written consent was secured from all patients.

### Immunohistochemistry

IHC was performed as previously described [28]. Briefly, antigen retrieval was conducted in the microwave for 10 min pretreatment in 1 mM citrate buffer, pH 9.0. Slides were incubated in anti-CUL4B antibody (1:200, cat no. #C995; Sigma, USA) or PAX8 (1:100, cat no. ab239363, Abcam) overnight at 4 °C. Two independent pathologists (H.G. and B.H.) then blindly evaluated the slides. CUL4B expression was scored by multiplying two parameters, including the percentage of positive cells (range from 0 to 4, 0 (0 %), 1 (1–25 %), 2 (26–50 %), 3 (51–75 %), and 4 (76–100 %)) and the staining intensity (range from 0 to 3). The final scores were defined as follows: 0 (negative), 1–3 (weak positive), 4–6 (moderate positive), and 8–12 (strong positive). For the analysis, we categorized negative and weakly positive tumors as the non-overexpression group, while moderately and strongly positive tumors were classified as the overexpression group.

### Cell lines and cell culture

Human thyroid follicular cell line Nthy-ori 3-1 was purchased from Guangzhou Jennio Biotech Co., Ltd (China). Human PTC cell lines IHH4, BHP10–3, and BCPAP were obtained from Wakayama University of Japan. The ATC cell lines 8305C and 8505C were kindly provided by Professor Zhiyan Liu. Nthy-ori 3-1, BHP10–3, and BCPAP cells were maintained in RPMI-1640 medium (Hyclone, USA), with BHP10–3 supplemented with 1 % l-glutamine and nonessential amino acids (Gibco, USA). IHH4, 8305C, and 8505C were grown in a 1:1 mixture of RPMI-1640 and DMEM medium (Gibco). All media contained 10 % fetal bovine serum (FBS, Gibco) and were incubated at 37 °C in a humidified atmosphere with 5 % CO2.

### Cell transfection

SiRNA transfection was performed according to the manufacturer's instructions using HiperFect transfection reagent (Qiagen, Germany). The CUL4B siRNA (sense strand: 5′-GGCAGCACUAUUGUAAUUATT-3′ and anti-sense strand: 5′-UAAUUACAAUAGUGCUGCCT-3′) was synthesized by Qiagen. A non-specific negative control siRNA (sense strand: 5′-UUCUCCGAACGUGUCACG-3′ and anti-sense strand: 5′-ACGUGACACGUUCGGAGAATT-3′) was also used. For overexpressing CUL4B, CUL4B and empty control plasmids were introduced into thyroid cells using Lipofectamine 2000 (Invitrogen, USA). For protein stability experiments, ubiquitination plasmids, ubiquitination K48R mutant plasmids, and ARID1A overexpression plasmids were introduced into thyroid or HEK293T cells using Lipofectamine 2000 (Invitrogen, USA).

### Cell viability assays

The treated cells were plated in 96-well plates at a density of 4000 cells per well and incubated for 12–18 h. Then, 20 µl of MTS Reagent (Promega Corporation, USA) was added to each well and incubated at 37 °C with 5 % CO2 for 1.5 h. Optical densities were recorded daily at 490 nm using a plate reader spectrophotometer (Bio-Rad Laboratories, Inc., USA).

### EdU incorporation

EdU (5-ethynyl-2′ -deoxyuridine) incorporation was detected using the Cell Proliferation Kit (Ribobio, China). After transfection, the cells were seeded into 96-well plates. After EdU labeling, the cells were treated with 16 Apollo reaction cocktails, stained with Hoechst 33,342 (5 mg/ml), and visualized under a fluorescence microscope (Olympus, Japan). The percentage of EdU-positive cells was defined as the proliferation rate. Data were obtained from three independent experiments.

### Colony formation assay

The treated cells were placed in 6-well plates at a density of 800 cells per well and grown in a medium with 10 % FBS. After 14 days of culture, cell colonies were fixed and stained with 0.5 % methylene blue (Sigma, UK) in ethanol, and the number of cell colonies with more than 50 cells was counted in each dish (colony formation rate = number of colonies in each dish/800).

### Cell migration, invasion and the wound-healing assay

Migratory and invasive cells were transferred into 8-mm-pore-size transwell inserts (Costar, USA) or Matrigel Invasion Chambers (BD, USA), respectively. They were counted in three randomly selected microscopic fields (×200) within 24 h after transfection for quantification. Each experiment was performed in triplicate. The treated cells were seeded into 12-well plates with a 40–50 % density and incubated at 37 °C in 5 % CO2 for 6–8 h. 10 µl pipet tips were used to scratch across the well.

### RNA extraction and quantitative real-time PCR (RT-qPCR)

Total RNA was extracted using Trizol reagents (Invitrogen) according to the manufacturer's protocol. Subsequently, the RNA was reverse-transcribed into cDNA with the First Strand cDNA Synthesis Kit (Toyobo, Japan). The resulting cDNA was then subjected to quantitative PCR to measure the mRNA levels of CUL4B, using a SYBR Green PCR kit (Roche, Swiss) and following the recommended cycling conditions. All primer sequences were listed in Table 1. Relative mRNA levels are calculated by a 2−ΔCt method. GAPDH was used as an endogenous control.

**Table 1:** Sequence of primers used in this study.

Gene	Forward	Reverse
**RT-qPCR**		
CUL4B	TGGAAGTTCATTTACCACCAGAGATG	TTCTGGTTTAACACACAGTGTCCTA
PAX8	TCAACCTCCCTATGGACAGCTG	GAGCCCATTGATGGAGTAGGTG
FOXE1	TCATCACCGAGCGCTTCCCGTT	GCGGCTGCATCGTGCATGTA
NKX2	CAGGACACCATGAGGAACAGCG	GCCATGTTCTTGCTCACGTCCC
NF1	GATGTAAAATGTCTTACAAG	CTGCCACCTGTTTGCGCACT
GAPDH	CACCATCTTCCAGGAGC	AGTGGTCTCCACGACGTA
**ChIP-PCR**		
PAX8–1	TAGGCCCACCCTAATGACCT	CTGGGTGGGGACTGAAACG
PAX8–2	AGCACCTACCATTGTGCCC	TTTCACGGACACCAACCTGT
PAX8–3	AACCCTGCCAAGTCAACCAG	CAGACGGTCACTGGAAGTGG

These primers were purchased from Biosune Biotechnology (Shanghai, China).

### Western-blot analysis

Western blotting was conducted following previously established methods [30]. The primary antibodies used were against CUL4B (1:2000; Sigma, RRID: AB_1,847,340), ARID1A (1:1000; Abcam, ab182560, RRID: AB_3,096,240), and ubiquitin (Ub) (1:1000; CST, 20326S), respectively. GAPDH (Santa Cruz, USA) was used as a negative control. Immunoreactivity was visualized using an enhanced chemiluminescence kit (Millipore, Darmstadt, Germany). The signals were detected with Rapid Step TM ECL Reagent (Millipore, USA).

### Immunofluorescence (ICC)

For immunofluorescence analysis, 4 μm tissue sections were fixed and stained with antibodies against CUL4B (1:200, Sigma, HPA011880, RRID: AB_1,847,340) and ARID1A (1:100, Abcam, ab182560, RRID: AB_3,096,240). The sections were then treated with fluorescent secondary antibodies, followed by DAPI staining for nucleus visualization. Finally, we used a confocal microscope for observation and image acquisition.

### Bioinformatics analysis

Datasets of GSE33630 and GSE29265 were downloaded from the Gene Expression Omnibus database (GEO, http://www.ncbi.nlm.nih.gov/geo/). CUL4B and ARID1A expression lever and clinical data were downloaded from the website of the Cancer Genome Atlas (TCGA, http://genome-cancer.ucsc.edu/).

### *In vivo* experimentation

All animal experiments were conducted in accordance with the experimental animal welfare and ethics regulations of Qilu Hospital, Shandong University. Male nude mice (nu/nu) were injected with 8505C-shNC or 8505C-shCUL4B cells (2×10^6) at 5 weeks of age. Once the tumors reached a specified volume, the mice were treated with either a control (DMSO), trametinib (3 mg/kg), dabrafenib (60 mg/kg), or a combination of both. Tumor growth was monitored every 2–5 days until the mice were sacrificed, with tumor size serving as the primary measure of treatment response. The animal study received approval from the Institutional Animal Care and Use Committee of Qilu Hospital, Shandong University (Document No ECSBMSSDU2021–1-61).

### Statistical analysis

Statistical analyses were carried out with GraphPad Prism 9 software (RRID: SCR_002798). Correlations were assessed using the Chi-Square Test or Fisher's exact test. Comparisons between groups were performed using the Student's *t*-test or Mann-Whitney test. The significance of correlations was analyzed through the Chi-square test or Pearson's correlation coefficient. For survival analysis, the Kaplan-Meier method was employed, and the log-rank test was used to determine differences. The prognostic relevance of various factors was evaluated with the Cox proportional hazards regression model. Recurrence-free survival (RFS) was defined as the duration from surgery until the first indication of recurrence. A p-value of less than 0.05 was deemed statistically significant.

## Results

### CUL4B is up-regulated and predicts poor prognosis in TC

**Fig. 1:**
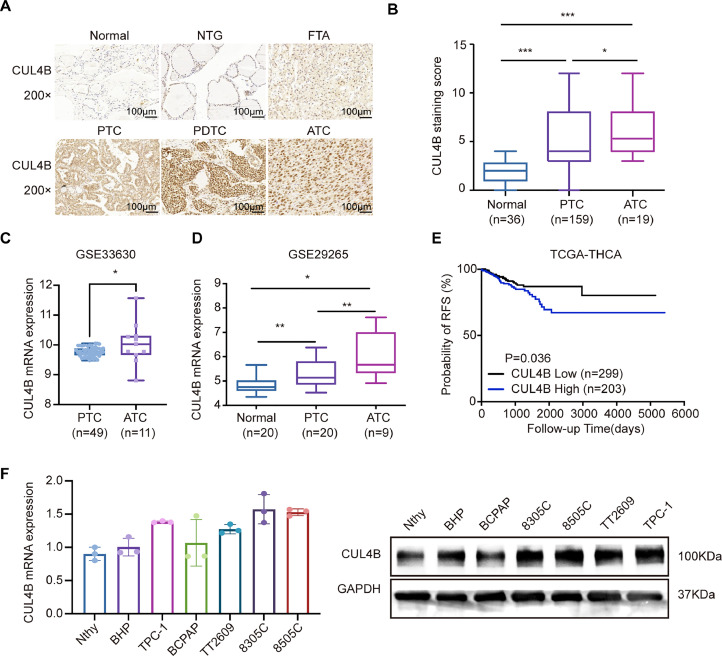
CUL4B is up-regulated and predicts recurrence in TC. **(A, B)**, Representative immunohistochemistry (IHC) images of CUL4B expression in TC (PV-9000 two-step method. Original magnification, ×200). **(C, D)**, The expression of CUL4B in TC as compared with normal thyroid tissues and ATC (Student's *t*-test. *P < 0.05, ***P < 0.001). **(E)**, The correlation between CUL4B expression and biochemical recurrence-free survival was assessed by Kaplan-Meier survival analysis (log-rank test). **(F)**, Analysis of CUL4B mRNA and protein expression in cell lines.

CUL4B was stained predominantly in the nucleus and cytoplasm using the IHC. Its staining intensity increased from benign lesions to malignancy. In detail, it is typically negative in normal thyroid tissues and multinodular goiter, weakly positive in follicular adenoma, moderately positive in PTC, and highly positive in PDTC and ATC (Fig. 1**A, B**). Statistical analysis showed a significantly up-regulated CUL4B level in PTC and ATC cases compared with adjacent normal tissues (Fig. 1**C, D**). CUL4B overexpression was identified in 40.9 % (65/159) of the PTC cases, whereas 59.1 % (94/159) cases were non-overexpression. Interestingly, IHC results showed exceptionally high CUL4B expression in aggressive PTC subtypes, including columnar cell, solid, tall cell, and hobnail PTC (*P* = 0.005, Table 2). CUL4B expression was also significantly associated with tumor diameter (*P* = 0.006, Table 2), lymph node metastasis (*P* = 0.028, Table 2), extrathyroid invasion (*P* = 0.006, Table 2), and the AJCC/TNM stage (*P* = 0.008, Table 2). Besides, CUL4B overexpression cases tend to show a high probability of loss of cellular cohesiveness, although without statistically significant (*P* = 0.053, Table 2). No association was identified between CUL4B expression and PTC patients' age, gender, tumor multifocality, and bilaterality (Table 2).

**Table 2:** Association of expression of CUL4B with clinicopathological parameters in PTC.

Characteristics	CUL4B		P value
	Low (%)	High (%)	
**Age(years)**			0.950
<55	69(50.0)	48(41.0)	
≥55	25(59.5)	17(40.5)	
**Gender**			0.521
Male	29(63.0)	17(37.0)	
Female	65(57.5)	48(42.5)	
**Histological types**			0.005*
Non-aggressive	83(64.3)	46(35.7)	
Aggressive	11(36.7)	19(63.3)	
**T stage**			0.006*
T1+T2	69(67.0)	34(33.0)	
T3+T4	25(44.6)	31(55.4)	
**Lymph node metastasis**#			0.028*
Negative	34(72.3)	13(27.7)	
Positive	56(53.3)	49(46.7)	
**Distant metastasis**			1
Negative	93(59.2)	64(40.8)	
Positive	1(50.0)	1(50.0)	
**UICC TNM stage**#			0.008*
*I*+II	88(63.3)	51(36.7)	
III+IV	6(31.6)	13(68.4)	
**Extrathyroidal invasion**			0.006*
Negative	74(66.1)	38(33.9)	
Positive	20(42.6)	27(57.4)	
**Multifocality**#			0.866
Negative	50(61.0)	32(39.0)	
Positive	43(62.3)	26(37.7)	
**Bilaterality**#			0.313
Negative	65(61.9)	40(38.1)	
Positive	27(71.1)	11(28.9)	
**Tumor spread**			0.113
Negative	88(61.1)	56(38.9)	
Positive	6(40.0)	9(60.0)	
**Loss of cellular cohesiveness**			0.053
Negative	58(65.9)	30(34.1)	
Positive	36(50.7)	35(49.3)	

# Values not available for all cases.

⁎ *P* < 0.05. χ2 or Fisher's exact test.

Based on the Kaplan-Meier analysis, the CUL4B overexpression group had a much greater recurrence rate than the CUL4B non-overexpression group (*P* = 0.0399, Fig. 1***E***). In Univariate Cox regression analysis, CUL4B overexpression was shown to be an independent prognostic predictor of PTC recurrence (HR=1.794, 95 % CI=1.014–3.173, *P* = 0.045). However, in multivariate analysis using stepwise backward entering of covariates (histological variants, multifocality, T stage, and lymph node metastasis), the prognostic value of CUL4B did not reach statistically significance (*P* = 0.172, Table 3). The expression of CUL4B in thyroid cell lines exhibited a pattern similar to that observed in TC patients through immunohistochemistry. Compared to Nthy-ori-3–1, CUL4B expression was significantly elevated in PTC, with the most pronounced increase observed in ATC (Fig. 1***F***).

**Table 3:** Univariate and multivariate analysis of variables associated with recurrence in PTC patients.

Parameter	Univariate analysis		Multivariate analysis	
	HR (95 %CI)	P	HR (95 %CI)	P
**CUL4B expression**	1.794(1.014–3.173)	0.045		0.172
**Histological variants**	2.307(1.241–4.288)	0.008		
**Multifocality**	2.159(1.173–3.975)	0.013	2.112(1.134–3.931)	0.018
**T stage**	1.812(1.002–3.276)	0.049		
**Lymphnode metastasis**	2.601(1.158–5.839)	0.021	2.736(1.144–6.543)	0.024

### CUL4B promotes migration, invasion, and proliferation of TC cells

**Fig. 2:**
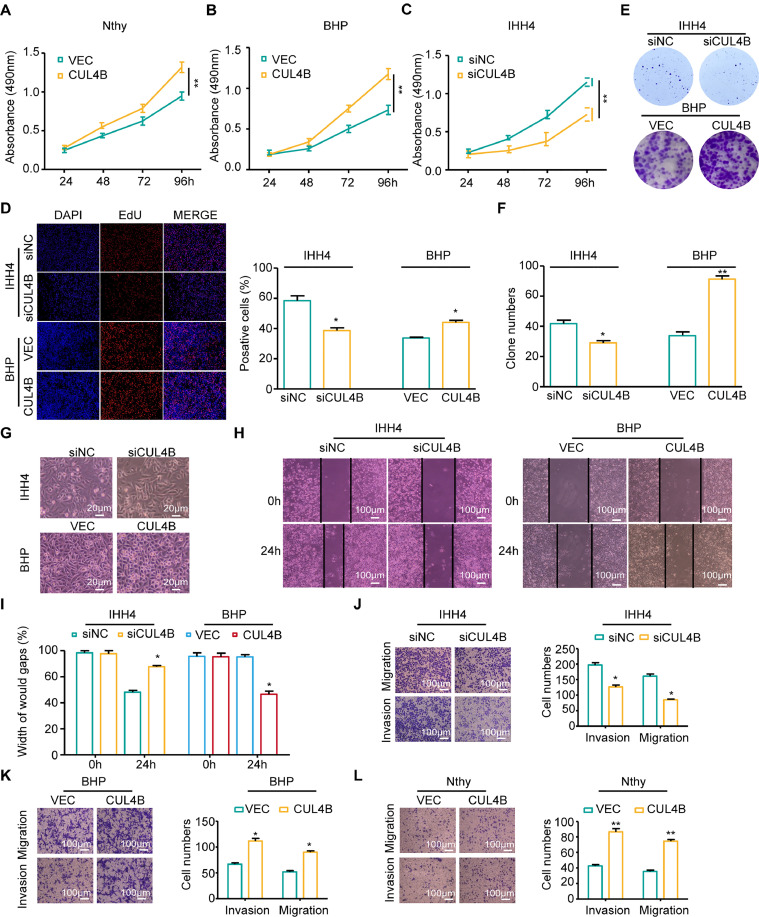
CUL4B promotes migration, invasion, and proliferation of TC cells. **(A–C)**, MTS assay showed that CUL4B enhances the proliferation of thyroid cells. **(D)**, EdU assay showed that CUL4B enhances the cell activity of PTC cells. **(E, F)**, CUL4B enhances the colony-forming efficiency of PTC cells. **(G)**, Morphological changes before and after CUL4B transfection. **(H, I)**, CUL4B enhances the thyroid cells' migratory capacity in wound-healing assay. **(J–L)**, CUL4B enhances thyroid cells' migratory and invasive capacity in a transwell assay. Data were analyzed using Student's *t*-test, *P < 0.05, **P < 0.01.

Transfection efficiency was validated, and then biological roles were investigated in IHH4, BHP10–3, and Nthy-ori 3–1 cells. MTS and EdU assays showed that down-regulation of CUL4B could reduce cell proliferation ability and cellular activities in IHH4 cells, and overexpression of CUL4B could promote proliferation and cellular activities in BHP10–3 and Nthy-ori 3-1 cells (Fig. 2***A–D***). Besides, down-regulation of CUL4B could reduce the number of colonies formed in IHH4 cells, and overexpression of CUL4B could promote the formation of colonies in BHP10–3 cells (Fig. 2***E, F***). Furthermore, compared to the control group, overexpression in TC cells results in a more rounded shape with increased morphological diversity, characteristic features of poorly differentiated TC cells (Fig. 2***G***). Wound-healing assay also confirmed that down-regulation of CUL4B could reduce cell migration ability in IHH4 cells, and CUL4B overexpression could promote BHP10–3 cell's migration ability (Fig. 2***H, I***). Transwell assays demonstrated that down-regulation of CUL4B significantly reduced cell migration and invasion abilities in IHH4 cells (Fig. 2***J***). In contrast, overexpression of CUL4B promoted migration and invasion abilities in BHP10–3 cells and Nthy-ori 3–1 cells (Fig. 2***K, L***). These results reveal that CUL4B promotes the proliferation, migration, and invasion abilities of thyroid cells.

### CUL4B deletion promotes TC differentiation

**Fig. 3:**
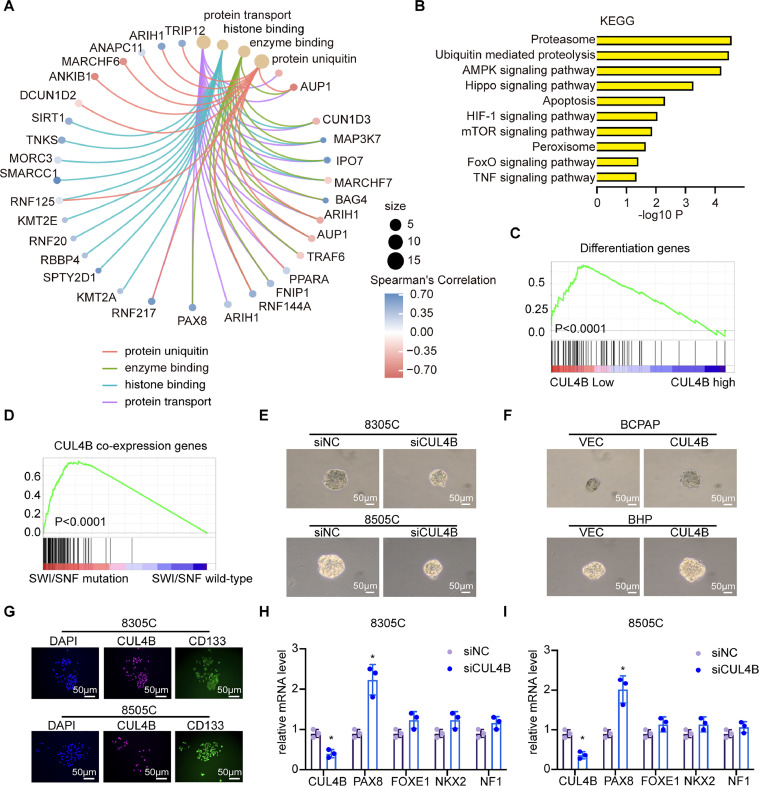
CUL4B deletion promotes TC differentiation. **(A)**, Gene sets significantly enriched in high-CUL4B tumors grouped accordingly to the original annotation in the MSigDB Database C2. **(B)**, KEGG terms of genes regulated by CUL4B. **(C-D)**, GSEA was carried out to examine the enrichment of the differentiation-decision genes and CUL4B co-expression gene sets. **(E-F)**, Sphere formation assay of TC cells. 8505C and 8305C cells were transiently transfected with siNC or siCUL4B. **(G)**, Cells were cultured for two weeks followed by staining with CD133 and CUL4B antibody and photography. **(H-I)**, The mRNA level of differentiation-decision genes, PAX8, FOXE1, NKX2 and NF1 determined by real time PCR in 8305C (H) and 8505C (I) cells. Data were analyzed using Student's *t*-test, *P < 0.05, **P < 0.01, ***P < 0.001.

To further investigate the molecular mechanism of CUL4B in repressing TC, we analyzed publicly available transcriptomic data from RNA-Seq studies of in CUL4B-depleted cells (Fig. 3***A***). As shown in Fig. 3***A***, according to Gene Ontology (GO) results, the differently expressed genes derived from CUL4B low-expression cells were enriched in cellular components such as the cell surface and plasma membrane and were functionally associated with focal adhesion. Interestingly, Kyoto Encyclopedia of Genes and Genomes (KEGG) analyses revealed that CUL4B low-expression significantly downregulated the ECM-receptor interaction signaling pathway. This pathway, crucial for intercellular communication, cell proliferation, adhesion, and migration, showed notable enhancement (Fig. 3***B***). Furthermore, GSEA analysis unveiled a notable enrichment of the differentiation signature linked to reduced CUL4B expression in TC cells (Fig. 3***C***). SWI/SNF mutations lead to thyroid dedifferentiation and tumor progression, while the loss of SWI/SNF promotes resistance to RAF/MEK inhibitor-based redifferentiation therapies in TC [31]. GSEA analysis revealed a significant enrichment of the CUL4B co-expression gene signature associated with SWI/SNF mutations in TC (Fig. 3***D***). We then analyzed the effects of CUL4B on self-renewal properties of TC cells. As shown in Fig. 3***E***, CUL4B loss significantly impaired secondary sphere formation in 8505C cells, whereas forced expression of CUL4B increased prostasphere initiation and growth in BCPAP cells (Fig. 3***F***). CD133 was essential for the proliferation and differentiation of tumor cells in cancer, identifying cancer stem cell-like populations that contributed to tumor growth and treatment resistance [32]. Due to its significance, we assessed the expression of CUL4B and CD133 in ATC cell lines and found that they were co-expressed (Fig. 3***G***). Subsequently, we knocked down CUL4B expression in ATC cells and assessed the expression of key differentiation-related genes. The results demonstrated that reduced CUL4B expression led to an increase in the expression of these critical differentiation-regulatory genes (Fig. 3***H, I***).

### CUL4B regulates the expression of arid1a through ubiquitination in TC

**Fig. 4:**
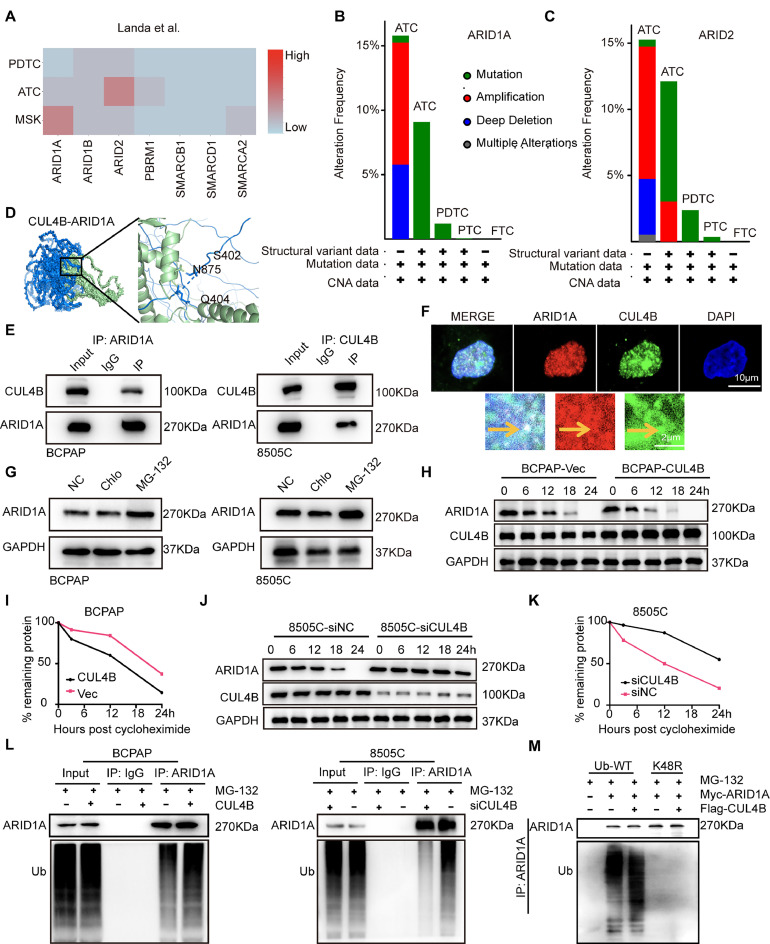
CUL4B interacts with and degrades the protein ARID1A. **(A)**, Frequency of SWI/SNF mutations in different cancer types. ATC has mutations in diverse SWI/SNF subunits. **(B, C)**, Frequency of ARID1A and ARID2 mutations in human PTC, PDTC, and ATC. **(D)**, Molecular docking analysis shows the interaction domain between CUL4B and ARID1A. **(E)**, Immunoblots of Co-IP assays showing interaction between CUL4B and ARID1A. Co-IP assays were performed using BCPAP and 8505C cell lysates. **(F)**, Immunofluorescence staining showing CUL4B (green) and ARID1A (red) localizations in 8505C cell. All pictures were imaged using a confocal microscope. Representative images are shown with a 10 μm scale bar. Boxes represent regions in higher magnification. **(G)**, 8505C, and BCPAP cells were treated with vehicle, 200 μmol/L chloroquine, or 20 μmol/L MG132 for additional 24 h. ARID1A protein levels were detected by immunoblotting. Chlo, chloroquine. **(H-K)**, 8505C, and BCPAP cells were transfected with CUL4B siRNA or expression vector for 24 h. Cells were then treated with 10 μg/mL cycloheximide and collected at 0, 6, 12, 18, and 24 h. ARID1A and CUL4B protein levels were determined by immunoblotting and the densitometry of ARID1A protein bands was normalized to GAPDH from triplicate experiments (I and J). **(L)**, 8505C, and BCPAP cells were transfected with CUL4B siRNA or expression vector for 24 h. Cells were then treated with 5 μmol/L MG-132 for 24 h, and subjected to immunoprecipitation using anti- ARID1A or rabbit IgG control antibody and immunoblotting using anti-ubiquitin antibody. **(M)**, H293T cells wes transfected with CUL4B, ARID1A, Ub-wild type or Ub-K48R expression vector for 24 h. Cells were then treated with 5 μmol/L MG-132 for 24 h, and subjected to immunoprecipitation using anti- ARID1A or rabbit IgG control antibody and immunoblotting using anti-ubiquitin antibody.

Genes encoding SWI/SNF subunits were mutated in nearly 25 % of cancers [5]. SWI/SNF mutations occur at a high frequency in TC, primarily involving the inactivation of SWI/SNF subunits ARID1A, ARID1B, ARID2, and PMRM1, which were found in several aggressive cancer types, including ATC (Fig. 4***A***). Bioinformatics analysis also revealed that the mutation frequencies of ARID1A and ARID2 gradually increase in PDTC and ATC compared to PTC (Fig. 4***B, C***). Next, we performed molecular docking analysis to identify the binding sites between CUL4B and these molecules. The results suggest that CUL4B binds most tightly with ARID1A (Fig. 4***D***). Co-immunoprecipitation (Co-IP) was performed in 8505C and BCPAP cells, and the results showed that there was an interaction between CUL4B and ARID1A (Fig. 4***E***). Immunofluorescence co-localization results showed that CUL4B and ARID1A are co-localized in 8505C cells, and the co-localization occurs within the nucleus (Fig. 4***F***). CUL4B was a member of the CRL family, the largest subtype of E3 ligases in mammals, targeted specific proteins for ubiquitination and subsequent degradation [33]. Thus, we hypothesized that CUL4B might affect the stability of the ARID1A protein. To explore this, we investigated the protein degradation pathway of ARID1A. The results indicated that ARID1A was primarily degraded through ubiquitination (Fig. 4***G***). Building on this, in BCPAP cells, overexpression of CUL4B increased the degradation of ARID1A protein compared to the control group. Similarly, in 8505C cells, reduced expression of CUL4B increased the stability of ARID1A protein (Fig. 4***H–K***). In continuation, ARID1A protein ubiquitination was decreased in 8505C cells when CUL4B was depleted. Conversely, in BCPAP cells, ARID1A protein ubiquitination was increased when CUL4B levels were elevated (Fig. 4***L***). Existing research has reported that the ubiquitin-mediated degradation of ARID1A was dependent on K48 linkage [34,35]. To further identify the ubiquitination sites of ARID1A, we performed mutagenesis of ubiquitination-related plasmids. The results showed that in the group transfected with the K48 point mutation of the ubiquitination plasmid, the ubiquitination level of ARID1A was markedly reduced (Fig. 4***M***). This indicates that the K48 point mutation significantly affects the polyubiquitination of ARID1A, suggesting that the K48 site plays a key role in the ubiquitination of ARID1A.

### CUL4B regulates PAX8 expression

**Fig. 5:**
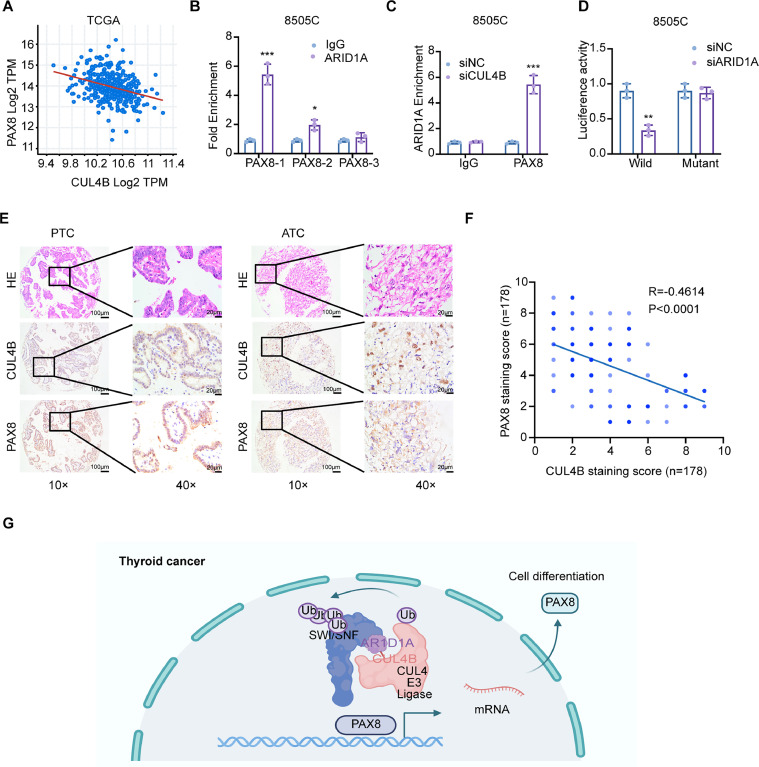
CUL4B regulates the expression of PAX8 through ARID1A in TC. **(A)**, Bioinformatics analysis using data from TCGA database showed that PAX8 expression was negatively correlated with CUL4B levels. **(B, C)**, ChIP-qPCR analysis of ARID1A recruitment onto the PAX8 promoter in 8505C cell. **(D)**, Luciferase reporters containing either wild-type or ARID1A binding site mutant PAX8 promoter were constructed. They were transfected in 8505C cell. Cells were also transfected with ARID1A siRNA. **(E-F)**, Representative H&E and IHC images of CUL4B and PAX8 expression of TC tissue with different intensity in Qilu cohort. **(G)**, A proposed schematic diagram illustrating the role of CUL4B in contributing to differentiation in the thyroid.

To determine whether PAX8 was a direct downstream target of CUL4B, we conducted a bioinformatics analysis using data from the TCGA database. Our findings revealed a negative correlation between PAX8 and CUL4B expression levels (Fig. 5***A***). Using JASPAR and the PROMO database, we identified a potential CUL4B binding site. ChIP-qPCR assays confirmed ARID1A recruitment to the PAX8 region in 8505C cell (Fig. 5***B***), which was decreased with CUL4B deletion (Fig. 5***C***). Luciferase reporter assay showed that ARID1A activated PAX8 wild-type but not the mutant promoter activity in 8505C cell (Fig. 5***D***). Subsequently, we investigated the relationship between PAX8 expression and CUL4B status. Remarkably, we observed a negative correlation between PAX8 and CUL4B expression in both clinical specimens of PTC and ATC (Fig. 5***E-F***). Building on these findings, our studies collectively demonstrated that CUL4B directly repressed PAX8 transcription through recruitment to the PAX8 promoter (Fig. 5***G***).

### CUL4B effects on the TC progression and sensitivity to chemotherapeutics

**Fig. 6:**
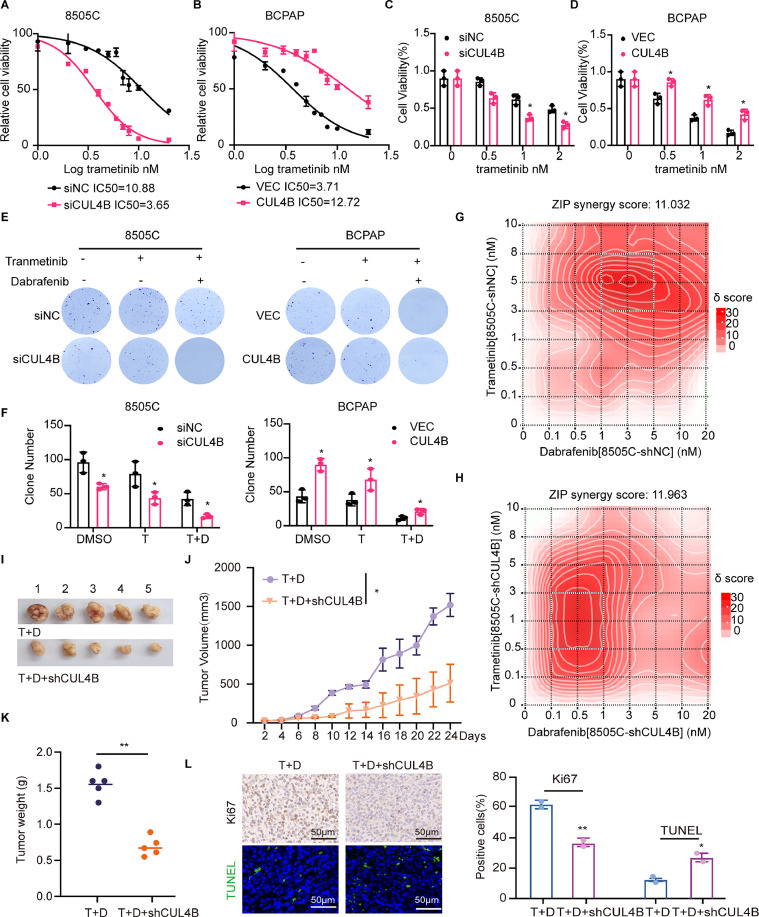
CUL4B deletion enhanced the chemosensitivity of TC cells. **(A-B)**, Trametinib IC50 detection by a MTT assay in siCUL4B-transfected 8505C and OE CUL4B transfected BCPAP cells. **(C, D)**, Cell viability of siCUL4B-transfected 8505C and OE CUL4B transfected BCPAP cells after treatment with trametinib in different concentration gradients for 48 h. **(E-F)**, A colony formation assay was performed in siCUL4B-transfected 8505C cells and CUL4B-overexpressing BCPAP cells to evaluate the impact of CUL4B on cellular proliferation. **(G, H)**, The 3D-plots showing effect on cell growth and drug synergism of trametinib and/or dabrafenib at varied concentrations in 8505CshNC and8505CshCUL4B cells. **(I)**, Representative images of subcutaneous xenografts in nude mice derived from shCUL4B transfected 8505C cells. **(J)**, Growth curves of the subcutaneous xenografts in each group. **(K)**, Analysis of the tumor weight of the xenografts in each group. **(L)**, IHC staining of Ki67 and immunofluorescence images showing TUNEL staining of prostate tumors on tumor slide from each group were shown. Scale bar represents 50 μm. T, Trametinib; D, Dabrafenib.

Considering the high expression of CUL4B in PDTC and ATC, as well as its influence on differentiation, we hypothesized that CUL4B might affect efficacy of MAPK inhibitors in TC, including trametinib and dabrafenib. As expected, CUL4B deletion enhanced the sensitivity of ATC cell lines 8505C to trametinib (IC50 = 5.36 nM) compared to control cells (IC50 = 10.88 nM) (Fig. 6***A***). CUL4B deletion enhanced the sensitivity of ATC cell lines 8505C to trametinib (IC50 = 5.36 nM) compared to control cells (IC50 = 10.88 nM). High expression of CUL4B increased the resistance of TC cells BCPAP to trametinib (Fig. 6***B***). These findings were further validated using MTT (Fig. 6***C-D***) and colony formation assays (Fig. 6***E, F***), indicating that CUL4B deletion can enhance the sensitivity of TC cells to trametinib treatment while also increasing the efficacy of its combination with dabrafenib (Fig. 6***G, H***). Finally, we investigated whether CUL4B influences the malignant behavior of TC cells *in vivo* (Fig. 6***I***). Furthermore, we found CUL4B could also significantly enhanced the sensitivity of mouse subcutaneous tumors to trametinib and dabrafenib treatment, resulting in tumor growth retardation (Fig. 6***J***). Compared to the control group, the tumor in the CUL4B low-expression group of mice was significantly reduced (Fig. 6***K***). To further assess tumor proliferation and apoptosis, we performed Ki67 and TUNEL staining. The results showed that, compared to the control group, the CUL4B low-expression group of mice had a reduced Ki67 index and an increased TUNEL signal, indicating enhanced apoptosis (Fig. 6***L***). The above data suggested that CUL4B could affect the efficacy of MAPK inhibitors in TC.

## Discussion

TC was characterized by a wide range of biological behaviors, from the relatively indolent PTC to the highly aggressive ATC. The progression from differentiated TC to ATC was marked by the loss of differentiation, which is a key factor driving the aggressive phenotype of ATC. Reprogramming cancer cell differentiation has shown therapeutic benefits across various disease contexts [36,37]. In TC, oncogenic BRAF suppresses genes critical for radioiodine sensitivity [38]. Mutations in SWI/SNF complex genes lead to a loss of chromatin accessibility at thyroid lineage specification genes in BRAF-mutant tumors, making them resistant to the redifferentiation effects of MAPK pathway inhibition [5,31].

Previously, ubiquitin-dependent pathways, especially those associated with CRL family proteins, were demonstrated to be vital for the quality control and regulation of multiprotein complexes [39,40]. In recent years, CUL4B has been reported to be up-regulated in a variety of tumors and involved in cancer proliferation and progression, acting as a tumor enhancer. Yin et al. found that RUNX2 recruits the NuRD (MTA1)/CRL4B complex, facilitating breast cancer progression and bone metastasis [41]. Earlier, we demonstrated that CUL4B promotes prostate cancer progression by establishing a positive feedback loop with SOX4, amplifying its oncogenic effects [29]. Additionally, CUL4B has been shown to promote gastric cancer invasion and metastasis through the upregulation of HER2, indicating its broad involvement in the regulation of multiple cancer types via distinct mechanisms [28]. In this study, we investigated the expression and biological functions of CUL4B in thyroid tumors for the first time. Our results revealed that CUL4B had the highest expression level in ATC, followed by PTC, and lowest in normal or benign thyroid tumors. Its overexpression was correlated with histological subtypes, tumor diameter, TNM stage, lymph node metastasis, and extrathyroid invasion. It is a sequential progression from indolent primary PTC to metastatic carcinoma, then to aggressive poorly differentiated carcinoma or ATC. So, our results indicated that CUL4B might promote thyroid cell malignant transformation. Besides, we found that CUL4B expression was closely correlated with PTC recurrence. Still, it was not an independent prognostic indicator in PTC, probably because the impact of CUL4B on recurrence was related to many other factors, such as histological subtypes and lymph node metastasis.

Among these, CUL4B has emerged as a crucial player in the transition from differentiated TC to undifferentiated ATC by inhibiting the expression of differentiation markers and promoting oncogenic pathways. CUL4B-based E3 ubiquitin ligase regulates mitosis and brain development by recruiting phospho-specific DCAF complexes [42]. Targeting DCAF5 can suppress SMARCB1-mutant cancer by stabilizing the SWI/SNF complex [43], underscoring the critical connection between CUL4B and the SWI/SNF complex. In this study, we demonstrated that CUL4B can ubiquitinate and degrade ARID1A, thereby competing with ARID1A for the transcriptional regulation of the key differentiation gene PAX8. The different ubiquitination sites on lysine residues serve distinct roles in protein regulation. Professor Zhang et al. found that mTORC1 promotes the polyubiquitination of ARID1A through K48 in hepatocellular carcinoma [34]. Similarly, Professor Beck et al. found that OTUD5 controlled neuroectodermal differentiation by cleaving K48-linked ubiquitin chains, emphasizing that the ubiquitination of ARID1A depended on K48 linkage [35]. We focused on investigating the role of the K48 site in the ubiquitination function of CUL4B. Our results demonstrated that the K48 point mutation significantly disrupted the polyubiquitination of ARID1A, highlighting the critical role of the K48 site in the ubiquitination of ARID1A in TC cells. Consequently, this competitive interaction further inhibits TC differentiation and promotes tumor progression. This competitive regulation plays a critical role in maintaining an undifferentiated, proliferative state within the tumor.

## Conclusion

Our findings show that CUL4B expression correlates with tumor progression and poor prognosis in TC. Mechanistically, CUL4B overexpression promotes TC progression and dedifferentiation *in vivo*. Notably, CUL4B induces dedifferentiation by ubiquitinating and degrading ARID1A in the SWI/SNF complex, reducing the differentiation marker PAX8, which contributes to ATC formation. Additionally, CUL4B silencing enhances TC cell sensitivity to MAPK inhibitors.

## Funding

This research was financially supported by the Natural Science Foundation of Shandong Province (ZR2023QH046) and the National Natural Science Foundation of China (Grant Nos. 81972416, 82172818, 82303905).

## Ethics declarations

Ethics approval and consent to participate

All procedures were conducted in compliance with relevant laws and institutional guidelines, and they received approval from the Medical Research Ethics Committee of Qilu Hospital, Shandong University. Approval for this study was granted by the Medical Research Ethics Committee of Qilu Hospital, Shandong University, in line with the principles of the Declaration of Helsinki (Document No. ECSBMSSDU2021–1-61 date: February 29, 2020). Informed written consent was secured from all patients. All animal experiments were performed in compliance with the Basel Declaration and received approval from the Institutional Animal Care and Use Committee of Qilu Hospital, Shandong University (Document No. ECSBMSSDU2021–1-61 date: February 29, 2020). Our animal experiments comply with regulations, ensuring that the size of solid tumors does not exceed 10 % of the animal's body weight. We confirm that the maximum tumor volume has not been exceeded.

## Data availability

All data and materials from this study are available from the corresponding author upon reasonable request.

## CRediT authorship contribution statement

**Haiyan Gu:** Writing – original draft, Resources, Formal analysis, Data curation, Conceptualization. **Bo Han:** Writing – review & editing, Project administration, Funding acquisition, Conceptualization. **Jing Hu:** Writing – review & editing, Visualization, Methodology. **Ping Liu:** Writing – original draft, Investigation, Formal analysis. **Wenyao Liu:** Writing – original draft, Visualization, Formal analysis. **Ying Qu:** Writing – original draft, Visualization, Formal analysis. **Lin Zhang:** Writing – original draft, Visualization, Software, Formal analysis. **Panpan Li:** Writing – original draft, Visualization, Investigation. **Gongzheng Wang:** Writing – original draft, Investigation, Data curation. **Zhiyan Liu:** Writing – review & editing, Supervision, Methodology. **Mei Qi:** Writing – review & editing, Supervision. **Feifei Sun:** Writing – original draft, Methodology, Investigation, Funding acquisition, Formal analysis, Data curation, Conceptualization.

## Declaration of competing interest

The authors declare that the research was conducted in the absence of any commercial or financial relationships that could be construed as a potential conflict of interest.

## Appendix Supplementary materials

Image, application 1
