# Supplementary figures and images for: CUL4B regulates thyroid cancer differentiation and treatment sensitivity by ubiquitinating ARID1A
Source: Transl Oncol. 2025 Apr 11;56:102389. doi: 10.1016/j.tranon.2025.102389 (PMC12013658; doi:10.1016/j.tranon.2025.102389)

**Figure 1 F**

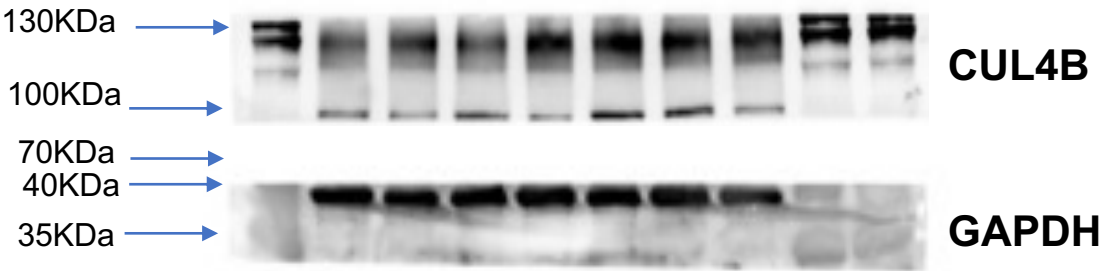

**Figure 4 E**

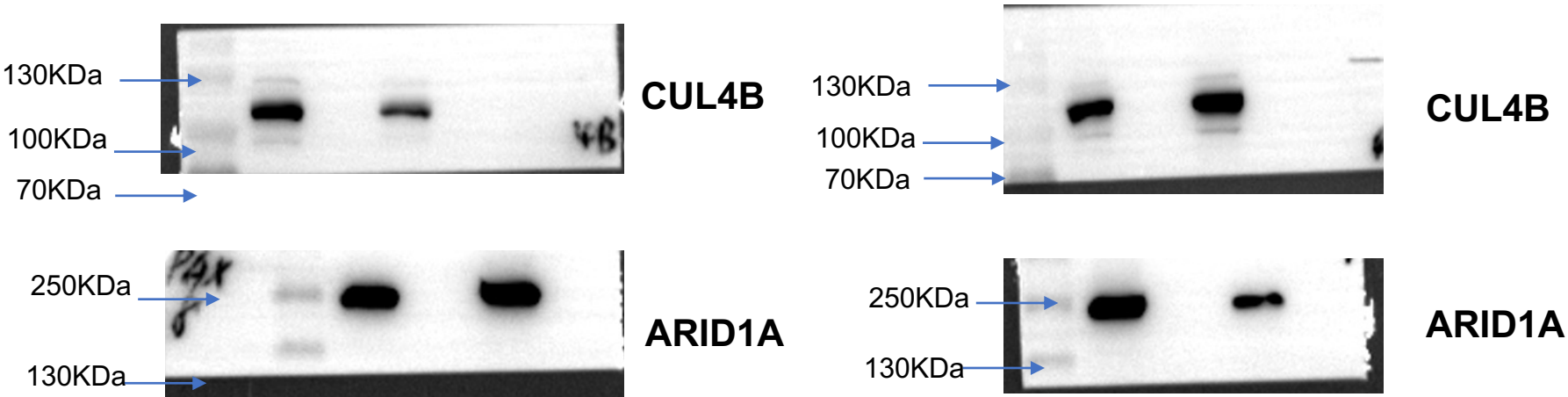

**Figure 4 G**

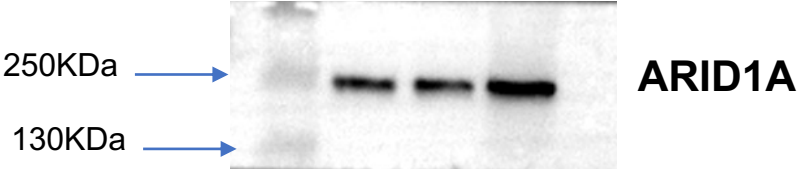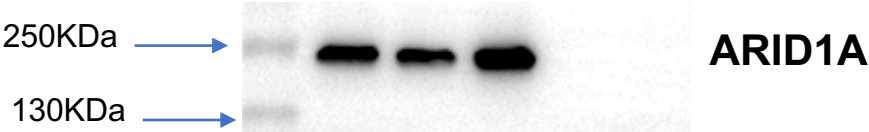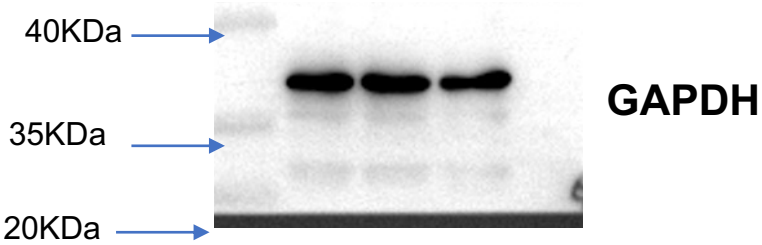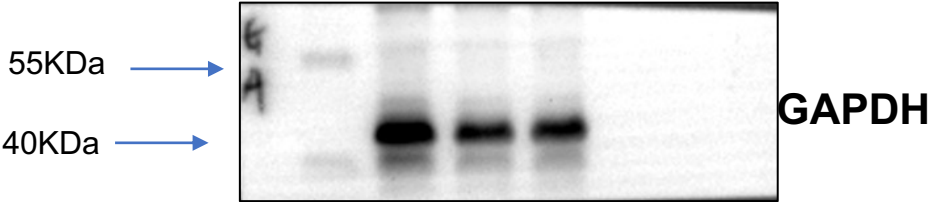

Figure 4 H J

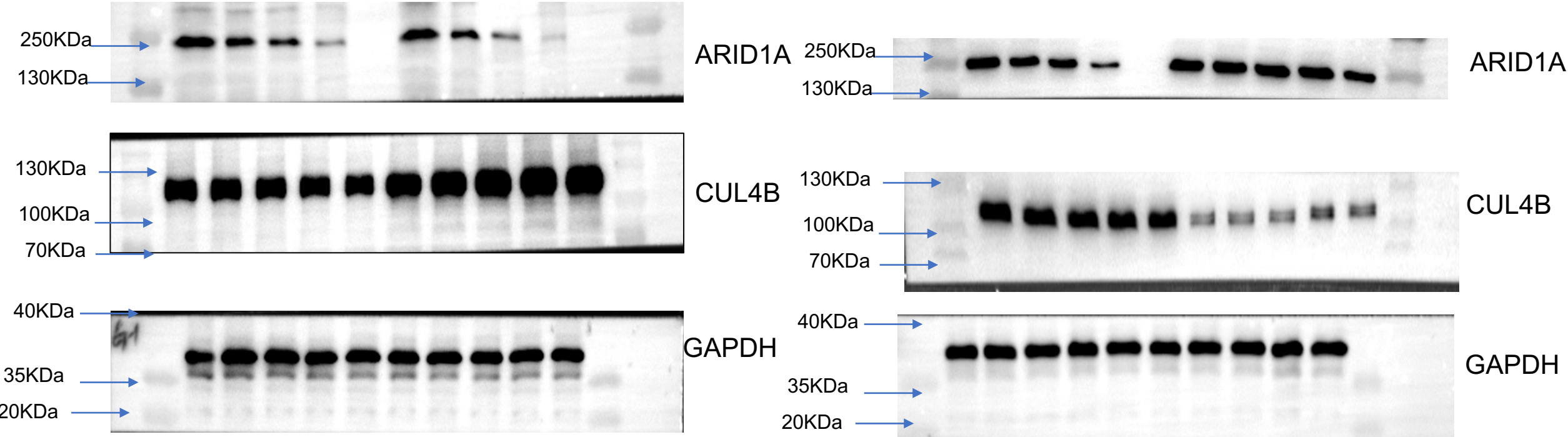

**Figure 4 L**

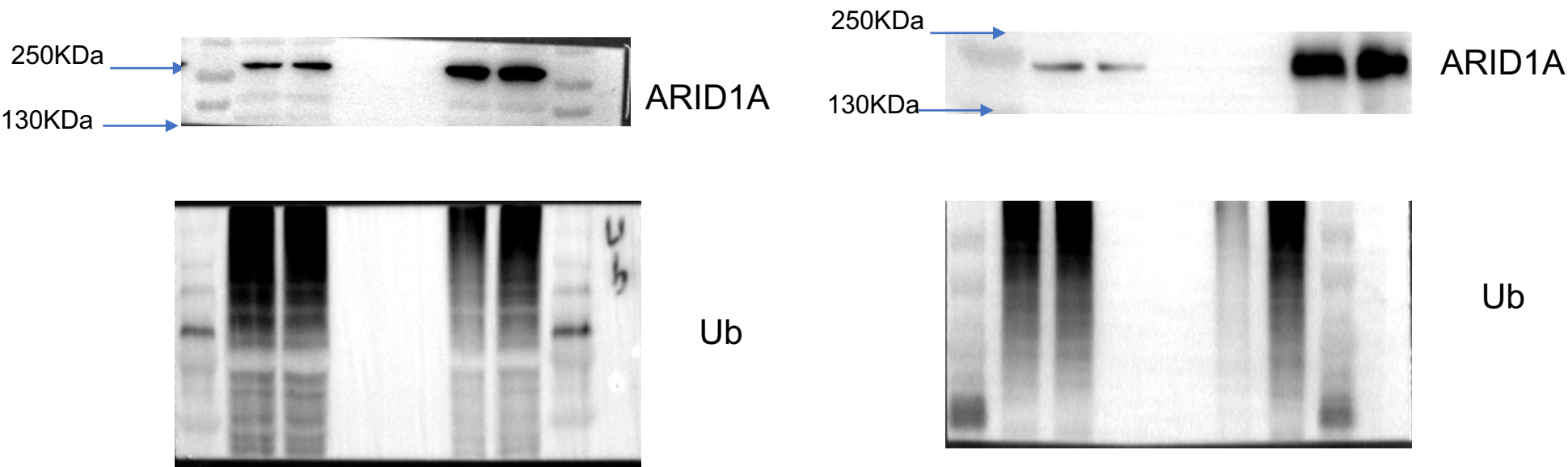

**Figure 4 M**

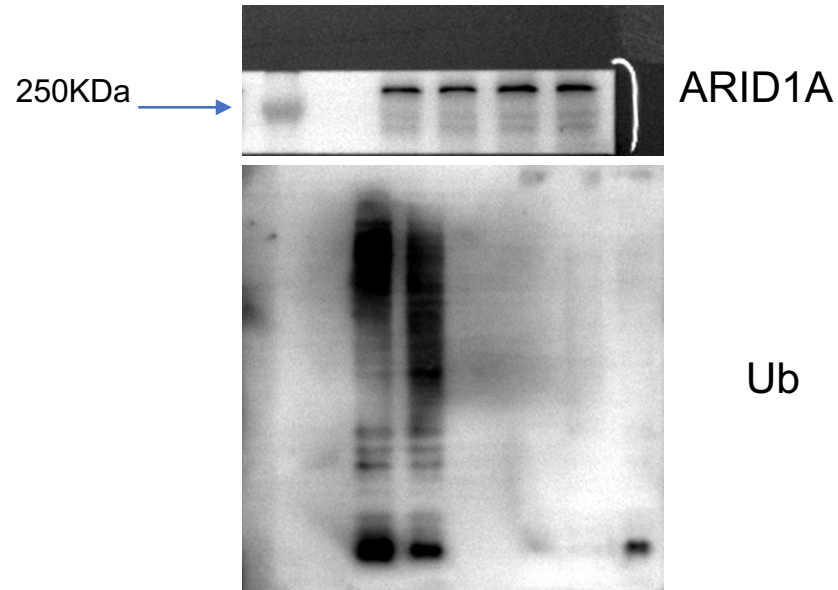

Supplement: Supplementary file 1 [file mmc1.pdf]
